# Observations on Mr. Bellott's Case of Hernia

**Published:** 1808-06

**Authors:** John Alder Bradley


### *To the Editors of the Medical and Physical Journal.*

Gentlemen,

In sending you the enclosed, I have to beg the favour of a place for them in your valuable Journal. If I have been so unfortunate as to differ in my opinion, with a professional Gentleman, I hope the importance of the subject will be my excuse. It has been my intention to avoid those illiberalities which too frequently disfigure papers of this nature; and as operative surgery is of so much importance to the community, so I have thought, that the most trifling deviations from established rule ought to excite our attention, as the statements and the practice do not appear to me consistent with a consideration of the structure structure of the parts, and as attempts at putting in execution the same appear to be attended with considerable difficulty and danger; I have at length taken the liberty of making those remarks upon the case, which I had hoped would before now have been made by a more able hand.

I am, &c.

JOHN ALDER BRADLEY.

*May* 15, 1808.

---

*Some Remarks on a Case of Incarcerated Femoral Hernia, published by Mr. A. Bellott, Surgeon, of Oldham; by John Alder Bradley, Surgeon, Stayley Bridge.*

THE importance of every thing brought before the public, in which the well-doing of mankind is concerned, is sufficient apology for our inquiry into the principles upon which the practice is built, and the comparative utility of the modes of practice laid down. The case upon which the following remarks are made, appeared in No. 101, of the Medical and Physical Journal. It is to the operative part I would wish more particularly to draw attention.

The case was one of femoral hernia, of long standing, which had not been returned for many years; the operation was judged necessary, proposed and put in execution. "The fasciæ and cellular membrane (we are told, p. 46), were cautiously divided, the whole length of the tumour, which exposed the hernial sac and ligamentum Poupartii, to which the sac was adhering: with some difficulty it was separated so as to admit the finger, and the ligament was dilated with Pott's bistoury introduced upon the finger, cutting obliquely downwards, and towards the os pubis." The adhesion here meant is certainly that adhesion which constantly takes place betwixt the sac and the portion of ligament which passes over it; an adhesion which takes place so early, that the herniary sac has only in a few instances been known to have been returned after its descent. This adhesion betwixt the sac and ligament was separated with some difficulty, so as to admit the finger under the ligament. Now, the closeness of the stricture in femoral hernia is so great, that it has been noticed by our best writers upon this disease. In the female, the closeness of the stricture is such, that Mr. Hey * says he could

---

* I have always found great difficulty in introducingthe smallest portion of the fore-finger into the femoral ring. Pract. Obs. p. 150.

could never, in any case, introduce more than the tip of the forefinger under the stricture; and this, which is so constant in the female, is still more close in the male, owing to the very extended insertion of the posterior thin margin of Poupart's ligament into the crest of the pubis.* When the finger can be introduced under the ligament in femoral hernia, the strangulation surely cannot be owing to the ligament; but whatever was the state of the ligament, we are told that it was divided by introducing Pott's bistoury upon the finger, and cutting obliquely downwards and towards the pubis. This is a direction for an incision meant to cut Poupart's ligament rather singular, since, to cut downwards and towards the pubis, would be inevitably to make an incision into the neck of the sac. It is a general rule in operating for the hernia, that the incision of the stricture in bubonocele shall be made upwards and outwards, in order to avoid the epigastric artery, which most commonly runs behind,† and on the inner side of the herniary sac; whilst in the merocele or crural hernia the incision should be made upwards and inwards, in order to avoid the same artery, which in this case runs on the outside of the sac,‡ about half an inch from its

---

I have always found the stricture particularly tight in this kind of hernia. C. Bell, Op. Surg. vol. 1, p. 275.

This, it may be observed, is a small outlet, strictly embraced by the crural vessels and epigastric artery on the outside, and by the firm insertion of the ligament into the os pubis on the other. C. Bell's Syst. Dissect. part 4. p. 100.

This kind of rupture in men, and the bubonocele in women, have a particularly narrow entrance. Lawrence's Treatise on Hernia, p. 30,

It is generally much more difficult to return the intestines into the cavity of the abdomen, than in the scrotal or umbilical hernia, on account of the narrowness of the neck of the sac. Monro on Crural Hernia, p. 84.

* This closeness of the stricture would very much surprise us, were we to consider as the crural arch that part which lies under Poupart's ligament, from its origin to its insertion. This is the view, as has justly been remarked by Mr. Hey, which seems to have been taken of it by Mr. Pott (vide Pott's Works, vol. 1, p. 133, et seq. edit. 1771.) To Gimbernat, Monro, and Cooper, we are chiefly indebted for more detailed accounts of the anatomy of the hernia. Dr. Monro first noticed the more extended insertion of the posterior thin foot of Poupart's ligament into the crista of the pubis in the male than in the female, and thus accounted for the rarity of femoral hernia in males, and the greater liability to strangulation. The anatomical description of these parts is particularly clear in the late work of Mr. Lawrence.

† Vide Treatise on Hernia, p. 106.

‡ Treatise on Hernia, p. 232.

its neck.* Mr. Pott is very vague in his directions for the incision of Poupart's ligament; he directs no precise part to be cut, but objects to an incision made directly upwards, and to one upwards and outwards.† Dr. Monro advises the edge of the knife to be turned towards the umbilicus.‡ Gimbernat separates the posterior thin foot of the ligament at its insertion, by drawing the knife horizontally along the body of the pubis; § and this appears to be the method generally put in practice. The posterior thin foot of the ligament can only be cut in Gimbernat's method, whilst, to cut any other part of the ligament, an incision would be required decidedly upwards. We may then venture to say, that in no part will it be possible to cut Poupart's ligament by an incision directed downwards and towards the pubis.

Poupart's ligament however, we are told, was divided, and afterwards the adhesions betwixt the sac and ligament were cautiously torn asunder all round. Mr. Bellott here means to say, "that the adhesions betwixt the ligament and the superior part of the sac, as it passes over it, were separated." These adhesions had been partially separated before, so as to admit the finger. The ligament does not surround the neck of the sac, but only passes over the superior part of the neck of the tumour. To separate the adhesions all round, would be to dissect away the herniary sac from the contiguous parts; a thing full of danger, on account of the size and importance of the blood vessels with which the sac lies in contact. Nor, indeed, would it be

* There are no general rules without some exception; the exceptions to this rule arise from the manner in which the hernia is protruded in bubonocele, and in which the obturator artery is given off in crural hernia. Rougemont says, that the situation of the spermatic chord will point out the course of the epigastric in bubonocele, " pour reconnoitre exactement la disposition de cette artere, *(l'Epigastrique)* il faut s'assurer de la position du cordon spermatique relativement au sac." See note to Monro on Crural Hernia, p. 62.

Mr. Thomson, of Edinburgh, has seen the obturator given off by the epigastric, and passing on the upper and then on the inner side of the neck of the sac in crural hernia, almost surrounding the neck of the sac. Mr Thomson found the obturator given off in this way, in six out of ten preparations. Vide Monro, on Crural Hernia, p. 67.

I have once seen the obturator artery given off by the trunk of the epigastric; indeed, it is not uncommon.

† Vide Pott's Works, vol. 1, edit. 1771.

‡ Vide Monro, on Crural Hernia, p. 93, and Appendix, p. 13, Extract from Dr. Monro, sen. Description of all the Bursæ Mucosæ.

§ New Method of Operating for Femoral Hernia, translated by Beddoes, 1795.

be an easy matter to separate so completely the adhesions betwixt the neck of the sac and the ligament, as is specified, " the portion of the sac which, lying under Poupart's ligament may be called the neck of the sac, is generally half an inch in length, and is frequently more."* The room which can be gained by a division of the ligament is seldom more than is sufficient to enable us to return the protruded parts, much less will a simple division of the ligament allow our tearing asunder adhesions betwixt it and the sac, of so great an extent. The room gained by making the incision in Gimbernat's manner is not sufficient to allow this; and it would only be possible by making so free an incision into the ligament as it passes over the neck of the sac, as might allow it to be freely dissected and raised up; a thing not only impossible, on account of the situation of the epigastric artery and the spermatic chord, but likewise on account of the strength and connections of the ligament itself. The only reason for dividing Poupart's ligament before opening the sac, is that of attempting to return the Hernia after the division, without opening the sac.† A return of the hernia was, however, not attempted at this time.

The next step of the operation consisted in " drawing down the tumour a little, which brought to view the mouth of the sac." We may in some measure be enabled to judge of the force necessary to execute this, when we reflect that the mouth of the sac is the very entrance from the cavity of the abdomen; that the neck of the sac is half an inch and more in length; that the sac adheres to the adjacent parts on every side; and that it would be necessary, when these adhesions were separated, to drag down a membrane, investing an extensive cavity, adhering on every side to the parietes of the cavity, and containing its viscera in its duplicatures; and as the force necessary to do this could only be exerted by taking hold of the herniary sac, so it will be apparent, that a force thus exerted would

* Vid. Treatise on Hernia, by Mr. Lawrence, p. 234.

† This is the mode of operating recommended by Dr. Monro, in his Treatise on the Bursæ Mucosæ; the reason chiefly insisted on is, the hindering the exposure of the contents of the hernia to the atmospheric air. A better reason for adopting it in femoral hernia appears to me to be, the less danger which would accrue to the obturator artery, when given off by the trunk of the epigastric, when the ligament alone is dilated and reduction attempted; when this lusus is present, it can scarcely fail being divided, by cutting at the same time the neck of the sac and the ligament.

would be very likely to produce a rupture of the sac or of its contents; an accident which has happened from the use of the taxis, and which would be very likely to happen when the sac is exposed, and where "the pulse is 140 or more, with stercoraceous vomiting, cold clammy sweats, and hiccup."

After the sac was pulled down, an attempt was made to get into it below the stricture, but in vain, "as a complete adhesion had taken place between the sac and its contents throughout the whole of the tumour;" an opening was then made above the mouth of the sac, into the cavity of the belly, and "I then introduced a bent probe, and broke down the adhesion to the bottom of the tumour." The only reason which can be given for dragging down the sac is, that of exposing the mouth so as to enable the operator to get into the sac from above downwards, as recommended by Monro; but the sac was pulled down, and the next thing done was attempting to get into the sac below the stricture, so that the motive for pulling down the sac does not appear. The possibility of breaking down adhesions with a probe is clear, when we are determined that the adhesions shall, vi et armis, be broken down; but the propriety of pushing a probe downwards into an herniary sac to break down adhesions, when we cannot perceive what part the probe is perforating, can only admit of a single opinion; and this more particularly when the vomiting is stercoraceous, the pulse more than 140, with cold clammy sweats, or, in other words, when we have every reason to conclude, from the symptoms, that the parts are verging to a state of gangrene. A complete adhesion had taken place, not only betwixt the sac and its contents, but between the intestine and the omentum. The case was one of chronic hernia; it had not been returned for many years; we are then founded in supposing, that the adhesions would furnish a very considerable resistance to the passage of a probe; and it will be clear, with such an universal adhesion, how very readily the probe would be pushed through the coats of the intestine. When we attend to the statement of the symptoms,* and compare them with the state of the intestine,

* There is, however, one symptom that comes on in this disease, which appears to be the immediate forerunner of death; and when it has taken place, I apprehend the operation will always be ineffectual; this is a general coldness attended with moisture over the surface of the body. — E. Home.

testine, we shall be struck with the want of relation in the one to the other; and when we compare the length of time which the incarceration existed, (for the space of eleven days) with the rapid resumption of the intestinal functions, we shall be more and more surprised. Although adhesions betwixt the sac, the intestine, and the omentum, had been separated to so great an extent, although the intestine was returned in a state of inflammation, yet not one single symptom is noticed after the operation.

With respect to the propriety of separating adhesions when so very extensive as they appear to have been here, there may be some question. Dr. Monro, senior,* thinks it most adviseable to leave them; whilst Mr. Lawrence† advises the destruction of every preternatural connection before the parts are returned. Mr. Pott, whose authority is of the highest consideration, wherever a practical point is to be determined, seems to take the midway; he tells us, if the adhesion be of parts of the intestine *inter se*, and such as prove very difficult to separate, it will be better to return the gut into the belly as it is, than to run the risk of producing inflammation by using force;‡ whilst, if the connection be with the sac it may be made free with. Mr. Lawrence§ says, that the "agglutination of the two sides of a fold of intestine has caused a sufficient obstacle to the passage of alimentary matter, to induce a fatal termination. When adhesions have taken place betwixt the intestine, the omentum, and the sac, and it is judged necessary to separate them, this is always done at the expense of the omentum and sac; and when the adhesions are very extensive and firm betwixt the intestine and omentum, and when at the same time the intestine is much indurated, the violence which we are under the necessity of doing to the omentum, in separating them, would appear a strong reason for completing the operation by an excision of the indurated portion of omentum.

Upon an attentive consideration of the statement of the situation in which Poupart's ligament was found, compared with the well known smallness of the crural arch, and

---

Home, Esq.—Cases and Obs. on Strangulated Hernia, Med. & Chirurg. Trans. vol. 2.—This state, noticed by Mr. Home, is what has been generally understood by the term, cold clammy sweat.

* Vide Appendix to a Treatise on Crural Hernia, p. 11.

† Treatise on Hernia, p. 153.

‡ Works, vol. 1st. p. 104. Edit. 1771.

§ Treatise, p. 153; for this Mr. Lawrence quotes Cooper.

and of the direction in which the incision was attempted to be made into the ligament, together with the necessity which was found for dragging down the tumour to expose the mouth of the sac, I have been inclined to doubt, whether in this case the ligament was cut at all. From reflecting upon the connexions of the sac, not only to the adjacent parts, but considering it as an elongation of the peritoneum, I must call in question the possibility of dragging down the sac, half an inch or more, so as to expose its mouth exterior to Poupart's ligament; and if we are disposed to grant its possibility, we are called upon to condemn its propriety. The danger which might accrue to the patient by breaking down the adhesions with a probe, has likewise been another subject which has called for comment.—If in these remarks, I have in any expression over-stepped the dignity of a scientific discussion by the warmth of expression, my only duty remains now to apologize; of this, however, I am not aware. As I have not the honour of knowing the narrator of the case, so my opinions have been guided by the established rules of practice, which, in the remarks, I have endeavoured to oppose to what appears innovations; the grounds of my opinions have been fairly laid down, and I hope that nothing has been asserted but from an evident deduction.——

"Si quid novisti rectus istis;
Candidus imperti: si non, his utere mecum."

*Horat. Epist.*

